# Mining Legacies and Environmental Injustice: Systematic Mining-Led LULC Change in the Witwatersrand Mining Belt, Gauteng, South Africa (1990–2020)

**DOI:** 10.1007/s00267-026-02543-9

**Published:** 2026-10-10

**Authors:** Samkelisiwe Khanyile, Wendy Tsoriyo, Phemelo Mahamuza

**Affiliations:** 1 Gauteng City-Region Observatory, University of Johannesburg, University of the Witwatersrand, Johannesburg, South Africa; 2 Africa Centre for Evidence, University of Johannesburg, Johannesburg, South Africa

**Keywords:** Land use land cover, LULC transitions, mining, sustainable development goals, environmental justice, Witwatersrand mining belt

## Abstract

Mining activities significantly shape landscapes and often pose sustainability and environmental justice challenges for surrounding communities. This paper examines land use and land cover (LULC) transitions and their sustainable development implications along the Witwatersrand mining belt in Gauteng, South Africa (1990–2020). Leveraging geospatial analysis, chi-square statistical testing, and spatial clustering, the findings highlight a critical tension between mining and sustainability efforts. While 54.53 km² of mining areas successfully transitioned to natural vegetation, landscape-scale degradation persisted, natural and planted vegetation declined, and barren land increased, with informal settlements also increasing and concentrating in mining-adjacent areas. Statistical significance tests (χ² = 11,319,251.69, *p* < 0.001; Cramér’s V = 0.60) indicate that 97.9% of transitions are systematic rather than random, and that they concentrate in previously disadvantaged communities. Mining land was also converted to residential areas despite regulatory protections and buffer zones, indicating a policy and implementation gap. This study highlights the crucial role of LULC assessments in post-mining landscape management, concluding that practical mining sustainability requires an integrated approach of stronger regulation, affordable housing, participatory rehabilitation planning, and accountability mechanisms grounded in quantitative evidence of distributive and environmental justice.

## Introduction

The extractive nature of mining activities is known to fundamentally alter landscapes by exploiting minerals, often posing environmental and social challenges that persist long after mine closure. These impacts are almost always disproportionately distributed, usually concentrated near vulnerable communities. Understanding the effects of mining and mining-induced landscape transformation, therefore, requires careful examination of land use and land cover (LULC) change dynamics to reveal the spatial footprint of extraction activities, such as mining, and their environmental legacies.

Land cover refers to the observable biotic and abiotic characteristics of land, such as water, grasslands, bare soil, and forests, observed from satellite imagery (Rigolon and Flohr 2014). Land use describes how these land cover elements are modified and utilized regardless of their formal categorization (Atayi et al. 2016). Analyzing LULC change transition patterns over time reflects changes induced by both natural processes and human management decisions (Ballard and Hamann 2022; Hasan et al. 2020), presenting a vital indicator of sustainability and environmental justice in mining regions.

Questions on LULC change, sustainability, and justice are especially urgent in regions with a long-standing history of mining, which creates layered environmental legacies. Gauteng in South Africa is a case in point; its economy has historically been heavily dependent on mineral extraction, particularly of gold, which has underpinned the region’s infrastructure, economic development, and metropolitan growth. Despite mining’s declining economic role over the past three decades, its historical dominance of gold has created persistent spatial and inequality legacies that continue to influence current LULC, urban expansion and developmental trajectories in the province (Mubiwa and Annegarn 2013; Khanyile 2024). This is problematic, as LULC patterns also still reflect apartheid-era spatial planning and the co-location of disadvantaged communities with former mining zones, producing layered social and environmental vulnerabilities, and present urgent environmental justice challenges. Gauteng faces sustainability challenges, including environmental degradation and mine waste, which are further compounded by radioactive contamination (Musakwa and van Niekerk 2013; Hecht 2023). Rapid urbanization and population growth intensify these risks as residential areas, particularly informal settlements housing the most vulnerable groups, expand and encroach on former mine lands.

This paper examines LULC change along the Witwatersrand mining belt from 1990 to 2020. It utilizes satellite-derived land cover data and Geographic Information System (GIS)-based post-classification change detection, in Environmental Systems Research Institute (ESRI) ArcGIS Pro and RStudio, combined with chi-square statistical testing to address the following three key questions: (1) what are the spatial patterns and scale of LULC changes along the Witwatersrand mining belt over the past 30 years?; (2) how do these changes influence progress towards sustainable urban development in this mining region?; (3) what insights do these LULC transitions provide about environmental justice, particularly regarding the concentration of hazards and regulatory effectiveness in post-mining areas?

This study advances existing LULC research through three integrated methodological components: First, it employs statistical significance testing (chi-square analysis of transition matrices) to determine whether LULC changes are driven by systematic processes or random variation, indicating whether disparities arise from poor policy implementation or random settlement patterns, addressing a gap in prior research. Second, it applies spatial clustering analysis to identify local hotspots of environmental risk, moving beyond provincial-scale aggregation, to identify areas of concentration. Third, the analysis is grounded in environmental justice frameworks, using LULC patterns as evidence of distributional injustice and potential policy implementation failure. This study integrates both approaches by using statistically validated LULC transition patterns as quantitative evidence of environmental injustice. Together, these components create a replicable methodological approach for understanding mining-driven environmental injustice globally.

This paper is organized into six sections. Section one introduces the research context and questions. Section two reviews literature on mining, LULC change, and their links to sustainable and environmental justice; section three describes the study area and methods; section four presents the results; section five discusses the findings in relation to prior studies; and section six concludes with implications for addressing mining-related environmental injustice in Gauteng.

## Framing Literature: Concepts, Patterns, and Justice Linkages

LULC change research provides critical insights into how human activities transform physical landscapes and the social–ecological systems they support. This literature review is structured around four interconnected themes that frame the analysis of LULC change in the Witwatersrand mining belt. First, this literature review explores the inherent tensions between mining’s extractive nature and the principles of sustainable development. Second, it examines how mining-led LULC changes create distributional injustices for vulnerable communities. The paper also reviews GIS and remote sensing-based methodologies for quantifying these changes, emphasizing post-classification change detection and chi-square analysis as tools for assessing environmental justice.

### LULC Change in Mining Contexts

LULC changes and transitions are often permanent or difficult to reverse. Studies (Bridge 2004; Ololade et al. 2008; Sonter et al. 2014) show that mining leaves lasting legacy effects across different spatial and temporal scales, influencing land use decisions well after mining stops. These legacy effects appear through various transition pathways. Some former mining sites stay barren despite the existence of international best practices and national mining regulations, requiring progressive rehabilitation during the mine life cycle, with strict waste characterization, to ensure safe post-mining landscapes (International Council on Mining and Metals 2019; Ignatyeva et al. 2020). Others recover with vegetation following successful rehabilitation, while some are converted into residential areas, which indicates policy failure to prevent settlement on contaminated land. Understanding LULC transitions within these contexts, therefore, requires examining the nature and direction of these changes (which LULC is changing into which other LULC), the main drivers of change, and the consequences of these changes on natural and human communities in the immediate surroundings.

LULC transitions reveal spatial patterns that are rarely random. Vegetation is systematically replaced by mining infrastructure and built-up areas during active extraction (Mutimba et al. 2024; Lameck et al. 2025) and ecosystem reconstruction after mining is complex, requiring multidisciplinary approaches (Tibbett 2024). Yet the persistence of either barren land or the prevalence of informal settlements on former mining areas indicates policy gaps and suggests that integrated approaches are seldom realized in practice.

### A Paradox: Sustainable Development and Mining Practices

Mining activities conflict with the principles of sustainable development. Sustainable Development Goals (SDGs) 11 (Sustainable Cities and Communities) and 15 (Life on Land) emphasize the sustainability of cities and the protection of the environment. At the same time, mining often involves physical and chemical processes that contribute to environmental disturbances such as soil contamination and erosion (Liu et al. 2017), water contamination from acid mine drainage (AMD) (Culshaw et al. 2000; Liu et al. 2017; González-Martínez et al. 2019), degradation of plant life due to soil and air pollution, and adverse effects on crops, particularly in phosphorus-rich mining areas. Environmental justice research demonstrates that hazardous facilities are disproportionately located near marginalized populations (Bullard 1990), with mining activities representing a particularly severe form of environmental burden due to their spatial extent and temporal persistence (Keeling and Sandlos 2009; Verburg et al. 2011).

These challenges can often be traced back to the inadequate regulatory frameworks that have historically governed mining, producing persistent landscape legacies such as tailing storage facilities (TSFs) and waste dumps; loss of topsoil and greenery; mining scars and improperly closed mines and shafts; and socio-economic challenges for surrounding communities. Various stakeholders have worked to address environmental issues arising from mining practices, aiming to rehabilitate areas affected by mining through revegetation, land reshaping, waste relocation, and topsoil replacement to restore ecosystems, with some infrastructure converted to heritage, recreational, or industrial sites (Sibanda and Broadhurst 2018; Nuissl and Siedentop 2021). Nevertheless, it is critical to assess whether site-level rehabilitation is sufficient to reverse landscape-scale ecosystem loss or whether regional habitat degradation continues despite localized restoration efforts.

Addressing the sustainability challenges posed by mining activities involves a deeper understanding of the complex relationship between mining, urban processes and communities (Bainton 2020). It requires the integration of socio-economic considerations alongside environmental concerns from the onset of mining, rather than at advanced stages of the mining life cycle (Harrison and Zack 2012; Musakwa and van Niekerk 2013; Bainton 2020; Kengni and Nkosi 2023; Khanyile and Marais 2024). Yet SDG monitoring frequently overlooks the uneven allocation of environmental burdens, reinforcing the need to employ environmental justice frameworks and spatial analytical methods to accurately map the distribution of impacts (Chen et al. 2018; Nuissl and Siedentop 2021).

### Environmental and Social Justice in Mining Landscapes

Environmental justice refers to the equitable distribution of environmental benefits and burdens, as well as to the meaningful involvement of all people in the development, implementation, and enforcement of environmental decision-making, regardless of nationality, race, income, or social status (Schlosberg 2007; Walker 2012). Social justice extends to the broader societal structure, including access to resources, the recognition of identities, and full participation in governance, resulting in equitable lives (Kato-Huerta and Geneletti 2022). In the context of mining landscapes, these two dimensions converge, and the distribution of environmental hazards is often inseparable from the social processes that determine who lives near mines, who benefits from mining activities, and who bears the costs of their cessation.

LULC changes attributable to mining activity have immense environmental and social justice implications, particularly in regions where mining occurs near human settlements. Mining-related LULC changes lead to severe deforestation, land degradation and disruption of ecosystem services (Pretorius and Hattingh 2009; Kumar and Pandey 2013). For instance, in Asutifi North District, Ghana, LULC transitions reveal mining pits, barren areas, tailings dams, and unpaved roads replacing once-fertile land (Gbedzi et al. 2022). These changes have had cascading impacts contaminating local drinking water and posing food security and health challenges.

Poorer communities are also more likely to be exposed to environmental hazards and pollution (Boone et al. 2009; Rigolon and Flohr 2014; Hecht 2023), often bearing the brunt of environmental degradation, loss of essential resources, and health risks, which exacerbate inequalities and undermine their rights to a healthy environment (Liu et al. 2010; Bobbins 2013; Kumar and Pandey 2013; Limpitlaw and Briel 2014; González-Martínez et al. 2019; Gbedzi et al. 2022; Hecht 2023; Wang et al. 2023). Beyond environmental burdens, mining cessation introduces social ills such as crime and alters the social fabric of surrounding communities through forced relocation and displacement, loss of cultural heritage, family fragmentation and erosion of community trust as migrant labor often bypasses local economies (Marais 2013; Kivinen 2017; Esterhuysen et al. 2018; Mhlongo et al. 2019; Pelders and Nelson 2019; Seloa and Ngole-Jeme 2022; Owen et al. 2024), perpetuating socio-economic inequalities. Addressing these challenges is critical to achieving environmental justice and ensuring equitable access to natural resources for all affected populations.

To this end, understanding whether LULC patterns consistently reveal environmental inequalities helps to determine whether such disparities are due to policy failures or uncontrollable external factors, ensuring fair distribution of benefits and a more sustainable approach to resource management. Nevertheless, environmental justice frameworks have been predominantly applied in context in Northern and European contexts, with very few studies investigating the spatial manifestation of environmental and distributive injustices in mineralized urbanizations – areas that developed due to mining activity (Bryceson and MacKinnon 2012) – that are shaped by segregationist spatial planning.

### Geographic Information Systems (GIS) Applications in Land Cover and Land Use Change Studies for Investigating Mining Impacts

GIS is necessary for change assessments in mining contexts, enabling analysis and visualization of mining impacts (Mehta et al. 2024; Mutale and Qiang 2024). Many studies have highlighted the advantages of combining statistically rigorous approaches with methods used for analyzing LULC changes (Lu et al. 2004; Pontius et al. 2004; Rane et al. 2023).

#### Classification methods and change detection techniques

LULC change detection relies on the application of both remote sensing (RS), which provides multi-temporal satellite imagery and enables classification, and GIS, which can enable reference data collection and change analysis (Lillesand et al. 2015). Classification models for LULC change detection range from traditional maximum likelihood approaches (Otukei and Blaschke 2010) to advanced machine learning methods (Random Forest (RF), Support Vector Machines (SVM), commonly implemented in platforms such as Google Earth Engine, R and Python. These models have proven pivotal for detecting LULC changes in mining areas, with accuracies exceeding 85% (Asif et al. 2023; Rane et al. 2023). RF has been used to improve classification accuracy in mining regions (Ang et al. 2021), finding decreased primary vegetation and increased built-up areas, aiding socio-ecological management. Vegetation indices, while used to show high vegetation density, can also detect changes through image subtraction, which involves subtracting images of the same location at different times (Basommi et al. 2015). For instance, a study in Welkom, South Africa, applied five vegetation indices for LULC classification, achieving 88–96% accuracy, confirming their reliability for monitoring in extensive mining areas (Madasa et al. 2021). Post-classification change detection methods have also emerged as particularly effective for LULC change assessment, with recent studies confirming their superiority over pre-classification approaches (Gbedzi et al. 2022; Selmy et al. 2023; Seyam et al. 2023). This method is the most used, especially when comparing data from different sensors, and is considered highly accurate.

#### Transition matrix analysis and statistical validation

Post-classification change detection yields transition matrices that capture “from-to” information for each data cell or LULC class (Ahlqvist 2008; Narciso and Principe 2024). In coal mining regions, monitoring these transitions yields reliable data on the extent and rate of LULC changes, offering valuable insights for land management and policy development aimed at sustainable post-mining land use. Transition matrices provide the most comprehensive approach to understanding LULC dynamics, as they capture bidirectional conversions. They also quantify the magnitude of land cover transitions, which enables a detailed assessment of mining land pathways, and the generation of landscape metrics for evaluating effects of LULC changes (Pontius et al. 2004), which are vital to environmental justice (Selmy et al. 2023; Halder et al. 2024).

Previous studies have used spatial analysis or transition matrices to assess environmental justice (Owen et al. 2024; Pande et al. 2024). The chi-square analysis statistically assesses whether observed LULC changes are attributable to systematic processes or random variation (Akin and Berberoglu 2023; Pande et al. 2024). This methodology is particularly significant in environmental justice research, as it enables investigators to determine whether intentional factors or stochastic processes drive the clustering of environmental hazards near vulnerable communities. When transition matrices yield significant results (*p* < 0.001), they suggest that landscape changes are influenced by institutional, economic, or policy factors rather than chance occurrences (Pontius et al. 2004). Residuals from the chi-square test highlight LULC transitions with a z-score greater than (>) 2.0, indicating deviations such as vegetation loss, mining, or agricultural expansion, which can inform policy interventions. Spatial clustering indices further elucidate whether these changes are concentrated in specific areas, as clustered risks may require targeted community interventions, whereas dispersed patterns could reflect broader, underlying drivers.

The studies mentioned above demonstrate how GIS and remote sensing techniques provide essential tools for analyzing mining impacts and quantifying LULC change. When paired with statistical validation (chi-square tests, confidence intervals, spatial clustering) and policy-focused spatial analysis (regulatory buffer zones), GIS-based LULC assessments aid the identification of distributional injustices and policy gaps in mining-impacted areas. However, their application to environmental justice questions in mineralized urban contexts is limited and seldom grounded in quantitative landscape-scale evidence. This study seeks to apply these techniques to investigate LULC change across the Witwatersrand belt and assess progress towards the SDGs.

## Materials and Methods

This section outlines the study area, the datasets used, and the methodological approach applied to investigate LULC changes along the Witwatersrand mining belt spanning 30 years from 1990 to 2020. The analytical approach followed in this paper comprises LULC distribution, quantification of LULC changes, and statistical assessment of LULC changes in the study area.

### Research Study Area and Context

Gauteng province is the smallest province in South Africa, at just over 18,000 square kilometers (km²). It is located at an altitude of 1500 meters (m) above sea level, is geographically positioned within the Crocodile, Olifants, and Vaal Rivers watershed (Parker et al. 2017), and its soil tends to be sandy, owing to the grassland biome (Bredenkamp et al. 2006; Halder et al. 2024).

**Fig. 1:**
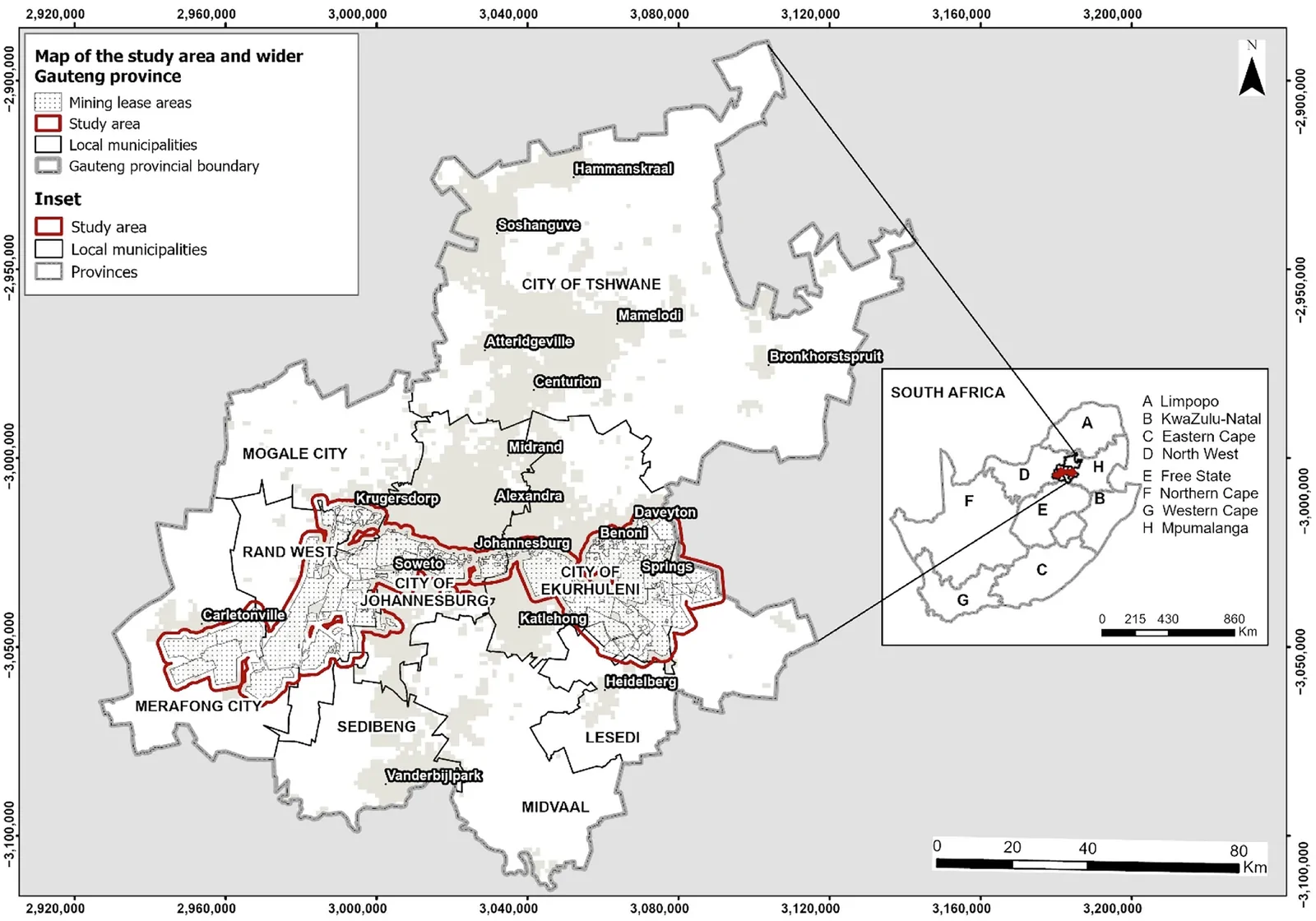
Map of the study area

The province is well known for its gold-bearing ridges of the Witwatersrand mining belt, discovered in 1886 (Harrison and Zack 2012) and is traversed by many mining lease areas (dotted areas in Fig. 1). The mining belt has over 270 Tailing Storage Facilities (TSFs) occupying 400 km^2^, some of which are radioactive due to co-occurrence with uranium (Bobbins 2013; Hecht 2023) – a factor central to understanding the environmental justice implications in the region. The result is that the Gauteng landscape is characterized by degraded landscapes with lowered environmental quality and socio-economic problems (Kivinen 2017) amid a bustling metropolitan region (Khanyile 2016).

#### Mining-impacted land, the policy environment and impacts in Gauteng, South Africa

In the post-apartheid period the governance of land and the environment in South Africa have shifted toward redress and equity (Patel 2014). This has been done through, amongst other things, the Minerals Act (MA) (No. 50) of 1991 (Republic of South Africa (RSA) 1991), regulating mineral rights and rehabilitation; the Constitution of the Republic of South Africa (RSA 1996), enshrining rights to an environment that does not harm one’s wellbeing; the National Environmental Management Act (NEMA) (No. 107) of 1998 (RSA 1998), mandating environmental authorizations, the Mineral and Petroleum Resources Development Act (MPRDA) (No. 28) of 2002 (RSA 2002), introducing mandatory rehabilitation provisions and mine closure certification; and the Spatial Planning and Land Use Management Act (SPLUMA) (No. 16) of 2013 (RSA 2013), guiding spatial planning. Before this, mining land was regulated by the Mining Rights Act (No. 20) of 1967 (RSA 1967), specifically between 1965 and 1980, facilitating the rehabilitation and allocation of previously mined land near settlement areas and the reallocation of previously mined land for previously disadvantaged population groups (Mubiwa and Annegarn 2013; Khanyile 2016).

However, despite these policy frameworks, Gauteng remains marred by persistent social and environmental challenges, with mining activities disrupting the natural environment and the region’s social fabric (Nzimande and Chauke 2012; Bobbins and Trangos 2018; Segerstedt and Abrahamsson 2019; Zou et al. 2019). Mining has contributed to air, water, and soil pollution, impacting communities such as Snake Park (Soweto), Tsakane, Rietvallei, Daveyton and Carletonville, which now face various health challenges. Additionally, studies (Lidzhegu and Palamuleni 2012) show that while land reform in South Africa has improved access for disadvantaged groups, it has also led to significant LULC changes, causing management and sustainability issues due to poor practices and limited capacity.

Responding to these challenges, the Gauteng Department of Agriculture and Rural Development (GDARD) established buffer zones around mine residue sites in 2003, further specifying minimum and maximum distances of 500 m to 1500 m under the 2012 Gauteng Pollution Buffers Policy, which was amended in 2017 and included in the Gauteng Provincial Environmental Framework (GPEMF) (RSA 2018). Additionally, GDARD identified 157 of 374 mine residue sites as radioactive, covering 220 km^2^. Rehabilitation has also been limited in this region, with only 25 km^2^ of waste able to be economically restored through revegetation, soil replacement, and reshaping, enabling use for industrial, agricultural, or residential purposes; however, such reclamation efforts can cause disruptions (Bobbins 2013).

This study proceeds from previous studies (Mubiwa and Annegarn 2013; Khanyile 2016; Katumba and Everatt 2021; Ballard and Hamann 2022) investigating LULC change in Gauteng. However, these studies have not employed environmental justice and spatial analysis frameworks, including statistical significance testing, to explore how LULC change transitions, particularly those linked to mining, can reflect alignment or deviation from efforts toward more sustainable and equitable development, and to assess their environmental injustice implications. For example, Mubiwa and Annegarn (2013) examine historical factors affecting spatial change in Gauteng, including mining, apartheid spatial planning, and the shifts in LULC from 1991 to 2009. They focus on urban development following the end of apartheid, revealing a highly dynamic exchange of LULC and competition for land among urbanization, cultivation, grasslands, and woodlands in the province. Khanyile (2016) demonstrates how historical mining activities in the region have profoundly shaped residential land use, while Katumba and Everatt (2021) and Ballard and Hamann (2022) show the unregulated and uncontrollable growth of formal and informal built-up areas at the core of the province. These studies report results at the provincial scale, missing identifying key local hotspots where environmental burdens are most concentrated and who bear disproportionate risks.

### Data Sources and Specifications

The study integrates multiple data sources, outlined in Table 1, to enable comprehensive spatial analysis. At the core of this paper’s analysis are the South African Land Cover (SANLC) (Department of Fisheries, Forestry and Environment (DFFE), 2022) datasets derived from satellite imagery (Landsat 5 Thematic Mapper (TM) for 1990 and Landsat 8 Operational Land Imager (OLI) for 2020) at a 30 m spatial resolution. This resolution is consistent with the regional scale of this analysis, which covers a ~2610.85 km^2^ of the Witwatersrand mining belt. High-resolution imagery was found to be costly for the landscape-level transitions under investigation in this study. The data were processed and classified by GeoTerra Image, on behalf of DFFE, and this study uses the post-classified products as secondary data.

**Table 1:** Data specifications used in the study

Data set name	Format	Sensor	Resolution	Source
SANLC 1990	Raster	Landsat 5 TM+	30 m	DFFE (2020)
SANLC 2020	Raster	Landsat 8 OLI	30 m	DFFE (2022)
Mining lease areas	Vector	NA	NA	Department of Mineral Resources (2011)
Gauteng Pollution buffer zones	Vector	NA	NA	GDARD (2017 in GPEMF 2018)
Gauteng provincial boundary	Vector	NA	NA	(MDB) (2016)
South African provincial boundaries	Vector	NA	NA	Municipal Demarcation Board MDB (2016)
South African local municipality boundaries	Vector	NA	NA	MDB (2016)

This study emphasizes analyzing transition patterns rather than classification accuracy; thus, it employs secondary data. The SANLC data utilized in this study have been validated and published, ensuring reproducibility. The data also uses areal data demarcating mining license areas, pollution buffers, and administrative boundaries.

### Data Preprocessing and Classification

All data were projected to the World Geodetic System (WGS) 1984 Universal Transverse Mercator (UTM) 35S projection system. SANLC data were clipped to the mining licenses and pollution buffers using the “Extract by Mask” tool. The original 73 (1990) and 72 (2020) classes of the SANLC data were collapsed into 12 comparable classes (see Supplementary Table S1) through hierarchical aggregation following Katumba and Everatt (2021). The SANLC data has primary, secondary and tertiary LULC classes in the attribute information. The SANLC 2020 data consolidates primary and secondary LULC categories, simplifying comparison. Tertiary-level classes sharing the same land use function were grouped into their corresponding secondary and tertiary categories. For example, “formal residential (trees)” was combined into “formal residence” for this study. This resulted in 12 LULC classes, while retaining thematic accuracy and enabling temporal analysis.

### Data Analysis and Methods

**Fig. 2:**
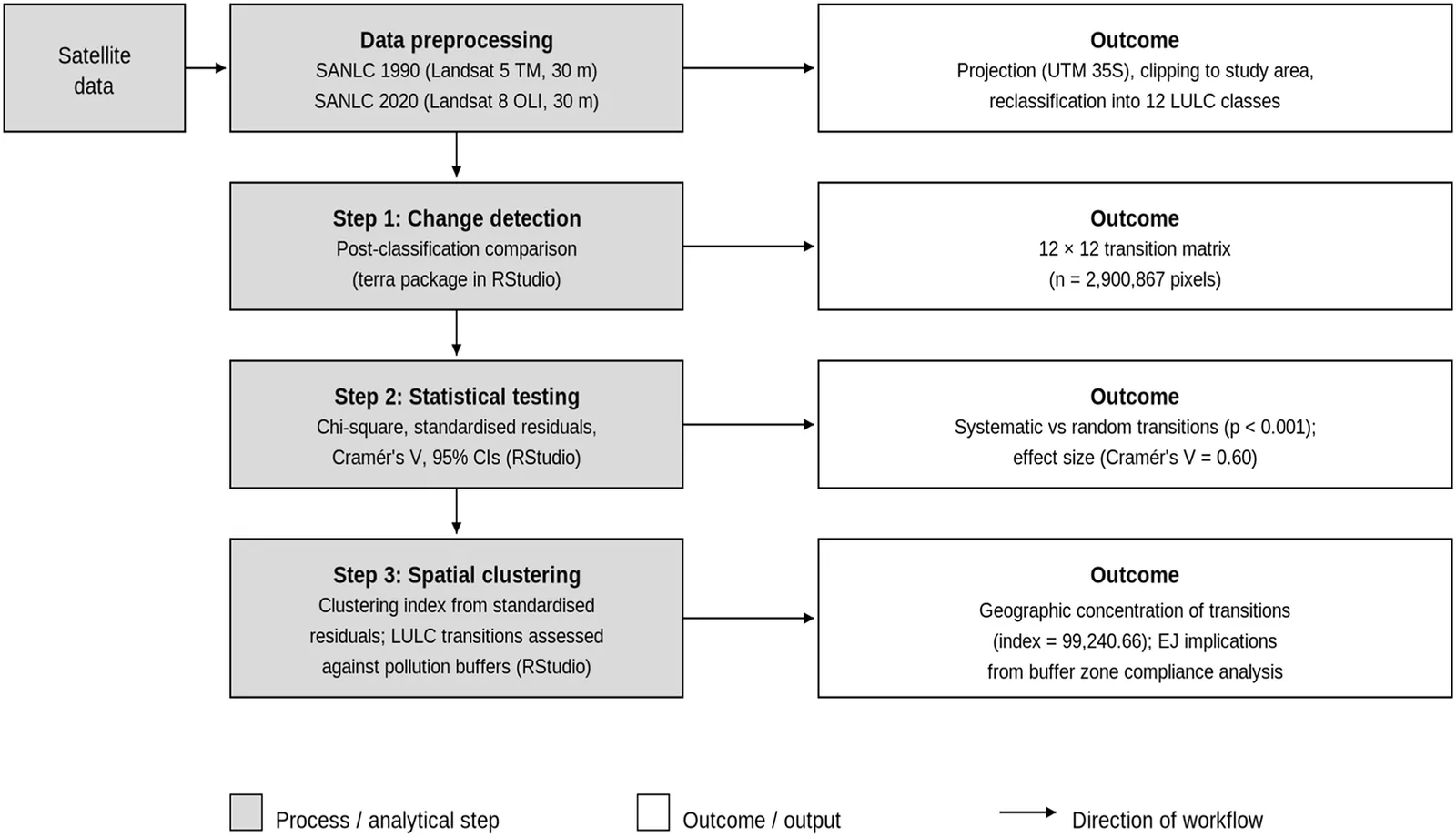
Flowchart of analytical workflow applied in this study

All data analysis was conducted in RStudio IDE on Posit Cloud and visualized in ESRI ArcGIS Pro 10.8 and Microsoft Excel. The analysis in this paper consisted of three steps, each aimed at deepening the understanding of LULC dynamics in this mining region. The analytical workflow is summarized in Fig. 2.

#### Change detection and transition matrix analysis

LULC in 1990 and 2020 was mapped to analyze and visualize its spatial distribution. This entailed standardizing the symbology across the SANLC 1990 and 2020 classes, enabling direct comparison of LULC patterns and distributions over a 30-year period. Thereafter, a post-classification change detection analysis was conducted, and a transition matrix was created. The terra package was used to cross-tabulate the 1990 and 2020 SANLC data to create a contingency table by recording pixel-level LULC changes. The transition matrix comprised rows of the 1990 LULC classes (i) and columns representing 2020 LULC classes (j), which were converted to area (in km^2^). Each cell contains the pixelated area transitioning between LULC classes. Persistence (where diagonal cells i = j) as well as transition areas were calculated. The LULC class persistence rates were calculated (persistent pixels/total baseline pixels) × 100 for each LULC class, showing which LULC classes were stable or had changed. Changes were also evaluated against the pollution buffers (500–1500 m) to assess compliance with environmental protection and environmental justice guidelines.

#### Statistical significance testing: Chi-square analysis and effect size

A chi-square analysis was used to test whether LULC changes were systematic or random, performed using the RStudio chisq.test function. The study’s focus is on the pattern and direction of transitions, which are best analyzed using the chi-square test of observed versus expected frequencies. With 2,900,867 pixels across a 12 × 12 transition matrix (144 cells), degrees of freedom = 121. Standardized residuals (z-scores) derived from the chi-square test reveal over- or under-represented transitions, with values exceeding residual >2.0 deemed significant at *p* < 0.05. Over-represented LULC transitions (z > +2.0) would occur more frequently than expected by chance, indicating the presence of systematic drivers of conversion. Under-represented transitions (z < −2.0) would occur less frequently than expected.

Additionally, effect size (Cramér’s V) was calculated to quantify the magnitude between the baseline (1990 LULC) and the final (2020 LULC). A range of 0 (no association), indicating minimal effect, to 1 (perfect association), indicating considerable practical significance; with the latter suggesting that transitions represent a substantial change in the LULC composition.

Transition proportions were estimated with 95% confidence intervals using the binomial method with the normal approximation, which is suitable for large samples (*n* = 2,900,867 pixels). Confidence intervals and margins of error were also utilized to estimate accuracy.

#### Spatial clustering index

To assess whether transitions were clustered or random, a spatial clustering index derived from standardized residuals normalized by cell count was used. This metric is derived directly from chi-square residuals and requires no additional spatial analysis software; it is purely attribute-based but enables identification of systematic geographic concentration. Higher values indicate stronger geographic clustering of transitions. For example, while random spatial distributions typically yield indices of 1000–5000, values >20,000 would indicate extreme clustering.

The results were then used to assess whether environmental burdens were disproportionately near vulnerable communities based on the geographic location of disadvantaged areas, to assess environmental injustice.

Additionally, this study investigates LULC changes relative to pollution buffers (500–1500 m) around mine residue areas. Transitions from mining or barren land to residential classes (formal and informal) or from mining to barren LULC within these buffers also indicate potential environmental injustice, with residential activity adjacent to potentially contaminated land.

## Results

This section presents the spatial, quantitative and statistical findings of LULC transitions in Gauteng’s mining-affected areas between 1990 and 2020. The results are presented progressively from spatial patterns and distributions to specific mining dynamics to reveal LULC dynamics in the mining region. Moreover, the findings are presented with a focus on progress towards meeting the SDGs, specifically for SDG 11 (Sustainable Cities and Communities) and SDG 15 (Life on Land) at the local scale.

### Spatial Distribution of LULC in 1990 and 2020

**Fig. 3:**
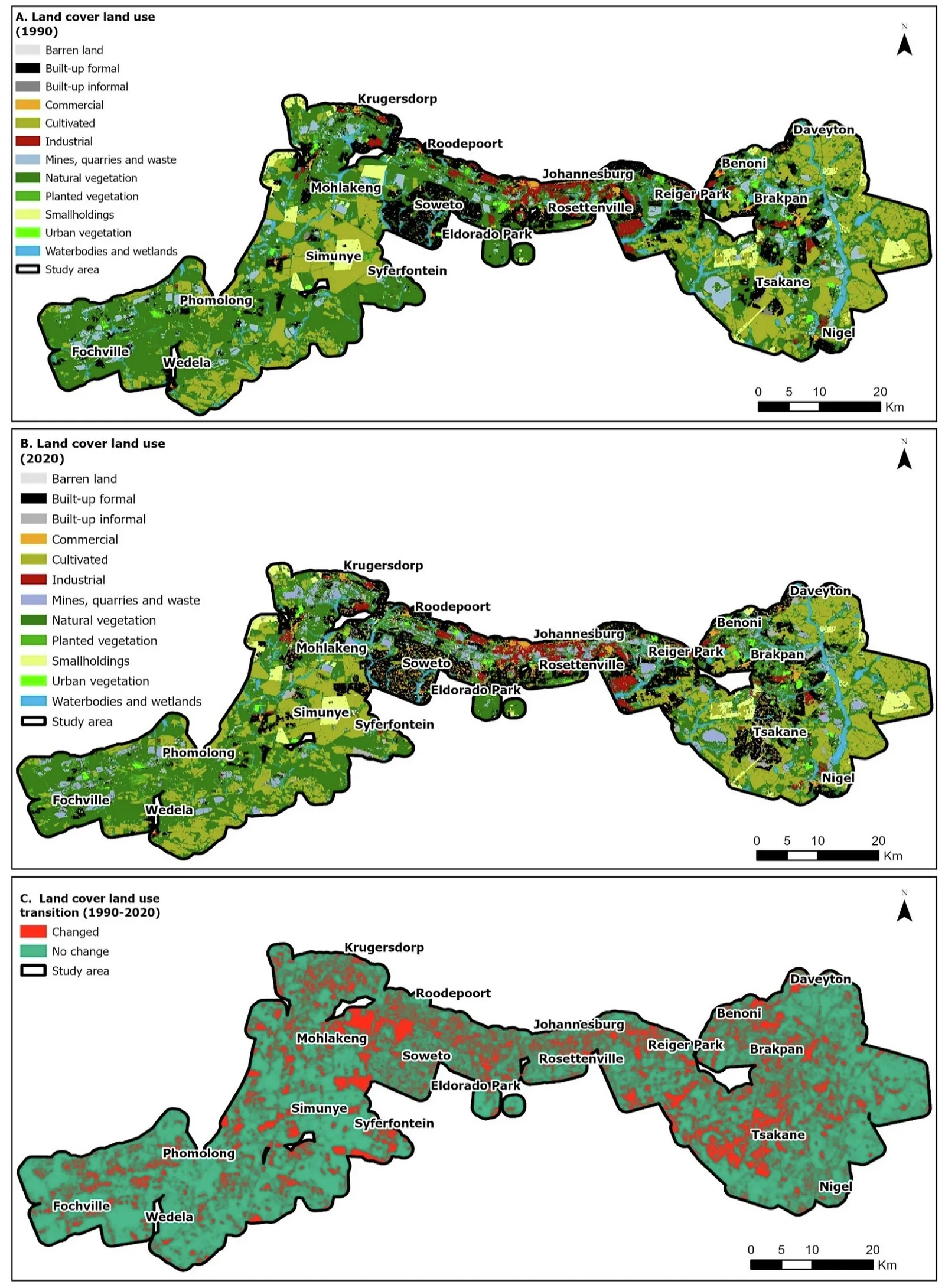
LULC along the Witwatersrand mining belt in 1990 panel (**A**), 2020 panel (**B**) and changes since 1990 in panel (**C**). Panel **C** shows unchanged areas in blue and changed areas in red, highlighting dynamic mining footprint patterns

Mapping the spatial distribution of LULC across the study period reveals significant landscape reorganization around the mining belt. Figure 3 illustrates LULC in 1990 (Fig. 3A) and 2020 (Fig. 3B), and changes in Fig. 3C. In 1990, the area featured mainly natural and planted vegetation, cultivation, smallholdings, and urban greenery, despite mining activities. By 2020, the LULC composition shifted towards urbanization, with less vegetation LULCs. Notable transformations occurred in Soweto, Tsakane, Simunye, and Mohlakeng - previously disadvantaged townships - where vegetation LULC was converted into commercial areas, formal and informal built-up areas, smallholdings, and various vegetation types. These changes are also illustrated in Fig. 3C. Peripheral areas, such as south of Mohlakeng, shifted to industrial LULCs. More importantly, mining areas remained near settlements in both 1990 and 2020, raising concerns about exposure to environmental hazards.

### Change Detection Analysis of LULC Change Since 1990

A change detection analysis assessed LULC changes from 1990 to 2020, revealing both the magnitude and direction of landscape transformation, in Table 2. Built-up classes grew steadily, driven by rapid urbanization. This expansion is the primary factor driving landscape change in the study area. The largest increase was in informal areas, which expanded by 42.91 km², a 847.89% increase. Formal areas grew by 60.74 km² (16.83%), and commercial areas nearly doubled with a 37.41 km² increase (116.47%). Barren land rose by 376.41%, indicating degradation. This increase highlights the combined impact of mining activities and poor rehabilitation, resulting in large areas unable to sustain vegetation or ecosystem services.

**Table 2:** LULC areal extent in 1990 and 2020, as well as the changes in the areal extent of LULC classes during this period

LULC class	1990 (km^2^)	1990 (%)	2020 (km^2^)	2020 (%)	Change (km^2^)	% change
Waterbodies and wetlands	134.45	5.15%	122.75	4.70%	−11.70	−8.70%
Natural vegetation	1104.94	42.32%	954.61	36.56%	−150.33	−13.61%
Cultivated	584.59	22.39%	613.76	23.51%	+29.17	+4.99%
Planted vegetation	62.10	2.38%	32.54	1.25%	−29.56	−47.60%
Mines, quarries and waste	144.25	5.52%	143.28	5.49%	−0.97	−0.67%
Barren land	5.80	0.22%	27.63	1.06%	+21.83	+376.41%
Commercial	32.07	1.23%	69.44	2.66%	+37.38	+116.55%
Industrial	65.83	2.52%	79.11	3.03%	+13.28	+20.17%
Built-up informal	5.05	0.19%	47.92	1.84%	+42.86	+848.04%
Built-up formal	360.02	13.79%	421.15	16.13%	+61.13	+16.98%
Smallholdings	90.34	3.46%	77.25	2.96%	−13.09	−14.49%
Urban vegetation	21.41	0.82%	21.41	0.82%	−0.00	−0.01%
**Total study area**	**2610.85**	**100%**	**2610.85**	**100%**	-	

Each LULC class’s area (km^2^) and percentage (%) for both years, along with the change in area (km^2^) and proportion

Conversely, some LULC classes decreased, notably planted vegetation (−29.56 km^2^, −47.6%), natural vegetation (−150.33 km^2^, −13.6%), waterbodies and wetlands (−11.70 km^2^, −8.7%), and smallholdings (−13.14 km^2^, −14.51%). These declines are especially concerning, considering the province’s pivotal position in the watershed and its reliance on scarce freshwater resources. An interesting observation is that mines, quarries, and waste declined slightly (−0.97 km^2^, −0.7%), suggesting sector consolidation and a reduction in active mining processes. Overall, these trends indicate that urban activity is intensifying at the expense of open spaces and green assets, thereby reducing the provision of ecosystem services.

### LULC Class Persistence and Stability

The analysis of which LULCs have changed or remained stable indicates constraining patterns (Table 3 and see Supplementary Table S2). LULC classes such as natural vegetation and waterbodies are converted outside their baselines, indicating degradation, while built-up classes show moderate to high persistence, indicating urbanization processes. Informal settlements, however, have a persistence rate of 45.42%, indicating that they have been actively changing since 1990.

**Table 3:** LULC class persistence rates: Percentage of each LULC class remaining unchanged (1990-2020)

LULC class	Area of persisting pixels (km^2^)	Persistence rate (%)	Area change pixels (km^2^)	Interpretation
Natural vegetation	765.06	69.24%	339.88	Moderate stability; substantial loss
Cultivated land	498.65	85.30%	85.94	High stability; agricultural resilience
Built-up formal	268.77	74.65%	91.25	Moderate stability; expansion zones
Mines, quarries, waste	81.82	56.72%	62.42	Low stability; rehabilitation/expansion
Built-up informal	2.30	45.42%	2.76	Highly dynamic; rapid expansion
Planted vegetation	17.29	27.84%	44.82	Unstable; converted to other uses

### Transition Pathways and Statistical Significance

Transition analysis uncovers pathways of land cover change, offering insights into landscape dynamics. Further, land-transition analysis in a transition matrix (Table 4) deepens understanding of change pathways. Where the LULC has remained unchanged from 1990, this is indicated in gray. The transition matrix shows that natural vegetation experienced a significant loss, with 339.88 km² converted to other uses: mainly mines, quarries, waste areas (41.55 km²), planted vegetation (10.22 km²), built-up areas (88.21 km²), and cultivated land (106.76 km²). This loss of natural vegetation to other uses suggests systemic pressure from competing land uses.

**Table 4:** Changes in the area (in km^2^) converted from other LULC since 1990

LULC in 1990	LULC changed into by 2020 (km^2^)											
	Waterbodies and wetlands	Natural vegetation	Cultivated	Planted vegetation	Mines, quarries and waste	Barren land	Commercial	Industrial	Built-up informal	Built-up formal	Smallholdings	Urban vegetation
Waterbodies and wetlands	**85.66**	37.84	4.16	0.35	1.21	0.88	0.11	0.26	1.09	2.78	0.04	0.07
Natural vegetation	27.36	**765.06**	106.76	10.23	41.55	19.16	6.65	12.35	25.28	88.21	0.77	1.57
Cultivated	1.48	26.54	**498.65**	0.31	5.6	1.63	1.87	1.04	9.72	37.35	0.26	0.13
Planted vegetation	0.93	20.75	0.31	**17.29**	2.16	1.15	0.83	0.92	1.36	9.55	2.48	4.38
Mines, quarries and waste	2.8	54.53	0.3	1.32	**81.82**	1.9	0.04	0.73	0.42	0.38	0.01	0.0
Barren land	0.22	3.14	0.32	0.07	0.45	**0.54**	0.04	0.25	0.11	0.65	0.0	0.01
Commercial	0.14	3.63	0.01	0.06	0.2	0.19	**24.2**	0.58	0.22	2.76	0.03	0.06
Industrial	0.24	6.5	0.03	0.15	1.61	0.64	2.15	**53.27**	0.38	0.77	0.07	0.03
Built-up informal	0.12	0.15	0.01	0.0	0.01	0.02	0.22	0.07	**2.3**	2.14	0.02	0.0
Built-up formal	2.2	32.27	0.6	1.43	8.4	1.14	32.58	8.65	2.69	**268.77**	0.36	0.93
Smallholdings	1.35	2.82	2.56	0.26	0.14	0.32	0.42	0.31	4.25	4.79	**73.13**	0.0
Urban vegetation	0.25	1.39	0.06	1.08	0.13	0.07	0.35	0.68	0.1	2.99	0.08	**14.23**

This table focuses only on LULC that changed and shows the area that changed from 1990 to 2020, along with what each highlighted LULC changed into. Diagonal cells (shaded in grey) represent land cover persistence

Built-up formal areas expanded, receiving conversions from natural vegetation (88.21 km²), smallholdings (4.79 km²), cultivated land (37.35 km²), and other categories, totaling substantial inflows that drove growth from 360.02 to 421.15 km². Along similar lines, cultivated land received multiple inflows from natural vegetation (106.76 km²), built-up informal (0.01 km²), smallholdings (2.56 km²), and other sources, increasing from 584.59 to 613.76 km². Conversely, some categories had minimal conversions: natural vegetation and waterbodies accounted for only 0.43 km², indicating that these systems are mainly sources of LULC conversion rather than destinations.

#### Statistical significance and systematic change direction

The effect size (Cramér’s V = 0.60) indicates large practical significance, meaning that most of these LULC transitions have changed the composition of the area. Standardized residuals also identified 29 over-represented and 112 under-represented transitions (see Supplementary Table S3). This suggests that LULC transitions are mainly characterized by class stability and where there is conversion it is driven by active conversion, rather than degradation. However, the under-representations also indicate that some transitions are occurring less than expected.

#### Main LULC transition pathways

The strongest over-representations were class persistence: natural vegetation to natural vegetation (residual = 989.89), cultivated to cultivated (1333.07), built-up formal to built-up formal (1083.84), all indicating LULC stability (Table 5). However, the major conversion pathways were natural vegetation to cultivated (residual = −476.39) and natural vegetation to built-up formal (residual = −323.18), both of which are under-represented, and occurring less frequently than chance would predict. The chi-square test of independence on the transition matrix yielded χ² = 11,319,251.69 (df = 121, *p* < 0.001), and 141 of 144 transitions were significant (97.9%), demonstrating that transitions were highly systematic rather than random.

**Table 5:** Top significant transitions (Chi-Square Analysis)

From LULC class	To LULC class	Area (km²)	Standard residual	Significance
Cultivated	Cultivated	498.65	1333.07	***
Natural vegetation	Natural vegetation	765.06	989.89	***
Built-up formal	Built-up formal	268.77	1083.84	***
Natural vegetation	Cultivated	106.76	−476.39	***
Natural vegetation	Built-up formal	88.21	−323.18	***

All transitions shown are statistically significant at *p* < 0.001 *** indicates significance

#### Spatial clustering and environmental justice implications of LULC transitions

To assess whether LULC transitions were concentrated in specific geographic areas or distributed randomly across the study area, a spatial clustering analysis was conducted. The spatial clustering index derived from standardized residuals of the chi-square test yielded a value of 99,240.66 (mean residual = 18.88), indicating strong spatial clustering of transitions. This high clustering index demonstrates that LULC changes are not randomly distributed across the Witwatersrand mining belt but are concentrated in specific geographic areas.

The concentrated geographic pattern of transitions has significant implications for environmental justice analysis. Rather than environmental impacts being evenly distributed, the clustering reveals that specific communities experience disproportionate exposure to multiple environmental stressors. Spatial mapping of changes and where they are located (Fig. 3C) shows pronounced changes in areas including Soweto, Tsakane, Simunye, and Daveyton - all historically marginalized townships with predominantly low-income residents. This geographic concentration suggests that environmental inequality in the mining region is not incidental but systematically structured by historical land-use planning legacies and current urban development patterns.

Mining areas slightly declined from 144.25 km² to 143.28 km² (−0.97 km², −0.7%), suggesting sector consolidation rather than expansion (Table 6 and see Supplementary Table S4). This decline probably indicates decreased mining activity and a more diverse land use as mining operations have declined over the past 30 years. 54.53 km² (37.8% of 1990 mining LULC) has been converted to natural vegetation, indicating active revegetation efforts. However, this is over-represented (z=8.19), indicating that conversion to natural vegetation occurs more frequently than predicted.

**Table 6:** Mining LULC key metrics. These metrics reflect both the restoration of mined areas and ongoing mining pressure on natural vegetation

Metric (km²)	Value	95% confidence interval	Interpretation
Mines, quarries, waste (1990)	144.25	–	Baseline extent
Mines, quarries, waste (2020)	143.28	142.59–143.96	Slight decline
Mines to Natural vegetation	54.53 (37.8% of mining change)	54.10–54.96	Positive rehabilitation; insufficient at the regional scale
Natural vegetation to Mines (expansion)	41.55	41.17-41.93	Ongoing mining pressure
Mines to Built-up	0.80	0.75–0.85	Policy failure: residential areas on contaminated land

At the same time, 41.55 km² of natural vegetation was actively converted to mining-related LULC, and 0.80 km² of mining areas were converted to built-up, risking residential placement near contaminated sites despite there being pollution buffers (500–1500 m). The expansion of informal settlements also disproportionately occurs in mining-adjacent areas. Together with the results of spatial clustering, this suggests that informal settlement growth is not randomly distributed.

These results collectively present three converging insights on environmental injustice. First, with the systematic nature of transitions (97.9% non-random). Second, these transitions are clustered in previously disadvantaged areas. Third, the conversion of mining land to residential use within pollution buffers raises concerns regarding their exposure to contaminated land. These patterns indicate that environmental burdens are structurally concentrated near vulnerable communities rather than incidentally distributed.

## Discussion

The findings reveal multidirectional LULC changes driven by mining, urbanization, and agriculture over three decades, with spatial clustering in historically disadvantaged areas. Statistical findings indicate that these transitions are not incidental. The findings from this paper are presented under four subheadings.

### Urbanization Processes in Mining-Affected Regions

The highly significant chi-square test result (χ² = 11,319,251.69, *p* < 0.001) demonstrates that land cover transitions were systematic rather than random. This indicates that urbanization patterns in the Witwatersrand were not haphazard but driven by underlying economic and institutional factors related to mining infrastructure and spatial planning legacies. The 33% expansion of built-up areas, particularly the 847.89% growth in informal settlements, suggests that mining-affected regions have become urbanization hotspots, with changes concentrated in previously disadvantaged areas such as Soweto, Tsakane, Simunye, and Daveyton (spatial clustering index = 99,240.66). These communities predominantly still marred by apartheid-era spatial planning, which located them in proximity to mining, face a fundamental spatial injustice: formally planned, serviced urban space is economically inaccessible to the poorest households, forcing low-income populations to occupy the highest-risk environmental zones (Mubiwa and Annegarn 2013). Environmental justice scholarship (Bullard 1990; Boone et al. 2009) suggests that such concentration in marginalized communities is evidence of injustice, especially when regulations fail to prevent exposure. Although there are regulations in place –such as the Constitution of South Africa (RSA 1996), noting everyone’s right to a clean, healthy and safe environment; NEMA (RSA 1998), mandating environmental authorizations; SPLUMA (RSA 2013), providing planning guidance and Republic of South Africa 2017 Pollution Buffers Policy (RSA 2018) enforcing buffer zones - there is a persistence of settlements on previously mined land. This indicates a gap between policy and implementation and raises both social and environmental justice concerns.

### Environmental Decline and Degradation

The analysis of LULC changes and transitions in the study area reveals three interconnected types of land degradation, which collectively undermine sustainability efforts. First, the analysis shows a 376.4% increase in barren land (+21.75 km²). This finding aligns with global mining studies (Limpitlaw and Briel 2014; Basommi et al. 2015), which show that barren land is a sign of environmental degradation and the loss of ecosystem function (Wang et al. 2023). This trend directly contradicts SDG 15’s emphasis on protecting and restoring terrestrial ecosystems and preventing land degradation. The continued and expanding barren land in mining areas indicates that current mine management is not restoring productive or ecological landscapes. Although economic remediation is possible, it often requires large investments that are not feasible in resource-limited contexts (Limpitlaw and Briel 2014; Basommi et al. 2015).

Second, the analysis shows a loss of vegetation. Natural vegetation decreased by 150.33 km² (−15.19%). Additionally, planted vegetation declined by 29.56 km² (−47.6%), representing the largest percentage loss among LULC categories. Smallholdings declined by −14.51%, reflecting shifts in agricultural practices within the province. These losses indicate environmental vulnerability and systematic degradation, as they reduce biodiversity and disrupt ecosystem balance, leading to soil erosion, degraded water quality, and habitat loss for wildlife (Culshaw et al. 2000; Ang et al. 2021). This study shows that vegetation loss tends to cluster in Gauteng’s township areas, such as Tsakane, Simunye, and Daveyton, north of Soweto, highlighting that environmental degradation mainly affects lower-income communities and raising concerns about environmental justice.

Third, waterbodies and wetlands declined by 12.12%, which is particularly troubling given Gauteng’s hydrological vulnerability due to being on a watershed divide and having limited water sources. Mining activities and urbanization are reducing the landscape’s capacity to retain, filter, and regulate water precisely when water demand is escalating. This loss of wetlands undermines ecosystem services critical for water security, biodiversity, and community resilience (Parker et al. 2017). The findings reveal an urgent sustainability concern where LULC changes are reducing natural water retention capacity while water demand increases. These findings indicate misalignment with regulatory frameworks such as the MPRDA (RSA 2002), which requires financial provisions for rehabilitation and closure and NEMA (RSA 1998).

### Quantifying Environmental Justice Along the Witwatersrand

Findings indicate that mine closure generates contradictory landscape outcomes (Table 6): while site-level rehabilitation is evident, the broader landscape experienced a net environmental loss, with declining natural and planted vegetation and waterbodies and increasing barren land. These findings are similar to those of studies (Wang et al. 2023) showing that mining damages ecosystems and recreational areas, creating barren landscapes with limited flora and fauna.

This paradox indicates a misalignment between regulatory compliance and environmental justice. On the one hand, it reveals that rehabilitation efforts, though positive, are localized to specific mine sites and insufficient to counteract landscape-scale habitat destruction or address environmental justice. Site-specific rehabilitation enables companies to demonstrate compliance and earn credits. However, environmental justice impacts are regional, with residents facing cumulative air pollution, ecosystem loss, and hazards. Studies (Cook 1971) note efforts to restore mined areas by planting vegetation and converting them into forests, providing ecological benefits such as air purification, erosion control, and climate regulation (Egoh et al. 2011). The findings, however, raise important questions about whether site-specific environmental remediation can meaningfully address cumulative ecological degradation in mining regions, particularly under ongoing pressure from urbanization. This limitation reflects international evidence indicating that point-source rehabilitation often cannot counteract cumulative regional impacts (Egoh et al. 2011; Festin et al. 2019). On the other hand, this stability in mining land suggests sector consolidation, where either (a) mining companies successfully defended property holdings through regulatory power, thereby monopolizing accessible urban land, or (b) mining actors strategically enabled settlement in buffer zones to monetize land assets. 0.80 km^2^ of mining LULC was converted into formal and informal built-up areas. Both scenarios reveal structural inequalities and environmental injustice: either formal land access is reserved for industrial uses while housing-poor populations are excluded, or mining companies profit from residential settlement in contaminated zones (Mubiwa and Annegarn 2013; Khanyile 2016). While justice scholars (Bullard 1990; Schlosberg 2007) frame environmental injustice as the disproportionate placement of hazards to marginalized communities, the Witwatersrand case extends this framework. It reveals that injustice is both spatial and temporal, with apartheid-era mining footprints continuing to structure both environmental exposure and development opportunities three decades after the regulatory regime that produced them was formally dismantled. This finding suggests that active rehabilitation is required alongside policy reform to resolve environmental inequalities.

### SDG Misalignment and Policy Implications

Examining LULC changes against SDG progress reveals mixed and concerning outcomes. SDG 11 (Sustainable Cities and Communities) aims for inclusive, safe, and sustainable human settlements. While urbanization occurred, it was predominantly informal and spatially concentrated in mining zones - the inverse of sustainable, planned development. SDG 15 (Life on Land) emphasizes protecting, restoring, and promoting sustainable use of terrestrial ecosystems. The 13.6% decline in natural vegetation, 47.6% loss of planted vegetation, and net habitat degradation directly contradict this goal. The findings demonstrate that regional development trajectories are not aligned with sustainability objectives and that mining-driven urbanization has prioritized economic extraction over environmental protection and social equity.

The LULC transitions (Tables 4–6) indicates that change is systematic (Cramér’s V = 0.60) and is shaped by institutional frameworks, infrastructure legacies, and policy choices. If transitions are systematic, they can potentially be deliberately influenced through targeted intervention. Yet findings from this study indicate a gap between current policy mechanisms, including buffer zones, rehabilitation and environmental mandates and practice, leading to ongoing habitat loss, settlement, and development on former mine land. This gap may reflect insufficient regulatory enforcement capacity, housing pressure overwhelming policy constraints, or limited alternative settlement locations. Closing this policy-implementation gap requires not only stronger enforcement but also integrated approaches that address housing shortages and provide communities with genuine alternatives to high-risk zones (Musakwa and van Niekerk 2013; Khanyile 2016). LULC change assessments, such as those applied in this study could also be integrated within these processes, enabling visualization and quantification of landscape-scale transitions beyond compliance at the site level, and therefore enabling evidence-based decision making. Specifically, a periodic LULC monitoring framework benchmarked against the GDARD’s 2017 Pollution Buffers Policy (RSA 2018) and MPRDA (RSA 2002) rehabilitation provisions would provide spatially explicit, enforceable accountability metrics for both government and industry. This would move oversight beyond self-reported site-level compliance toward independently verifiable, landscape-scale evidence.

These findings provide evidence of disproportionate exposure to the negative externalities of mining and environmental inequality; however, this study also carries some limitations. It relied on pre-classified low-resolution secondary SANLC data, which has 88–96% accuracy. This is an important consideration, as classification errors may compound during the change-detection process. Since the data depends on satellite imagery, it captures only surface impacts; issues such as subsurface contamination, groundwater effects, and community health outcomes are not captured, which requires future field research. The study relies on data from only two time-reference points which limit analysis of the rate of change. Furthermore, the lack of household-level socio-economic information limits insights into how land-use changes affect livelihoods and adaptive strategies within local populations. Finally, this study analyzes transitions but does not apply regression to model their drivers, which could be useful for showing causal inference. Despite these limitations, however, the findings from this paper offer robust, policy-relevant evidence of systemic patterns.

## Conclusions

While mining activity has declined over the past three decades, it continues to shape Gauteng’s development trajectories. This study examined mining-led LULC changes across the Witwatersrand mining belt from 1990 to 2020, a critical period coinciding with post-apartheid spatial restructuring, intensifying urbanization pressures, and various socio-economic challenges. The impacts of mining, be they environmental or social, are experienced unequally across the broader province. Therefore, given the location of Witwatersrand mining activities, understanding the impact of mining on the surrounding LULC is essential for assessing the sustainability of these activities and addressing environmental justice concerns arising from them.

This study’s findings reveal that mining impacts are systematic, suggesting that environmental inequities and injustices are institutional and policy-driven. The clustering index findings show LULC changes tend to concentrate in previously disadvantaged areas. Vulnerable groups are not randomly exposed but rather disproportionately exposed to polluted land, radioactive waste, and damaged ecosystems, despite new policies aimed at reducing such exposure due to apartheid-planning legacies. The study’s findings also reveal a paradox: while some mining areas transitioned to vegetation LULC classes, landscape-scale ecological decline persisted, with natural vegetation decreasing and barren land increasing. Additionally, some mining land was converted to residential settlements despite regulations prohibiting this. These changes highlight the environmental and socio-economic impacts of mining, emphasizing the need for sustainable practices and integrated urban planning. Findings suggest a gap between policy intent and implementation that continues to expose vulnerable groups.

Findings reveal systematic environmental injustice, indicating the need for more robust regulatory enforcement (with dedicated resources and meaningful penalties for violations), affordable housing in land use planning to alleviate pressure on contaminated land, participatory rehabilitation planning processes centering community voices and accountability mechanisms, such as landscape-scale transitions (empirically supported through spatial and statistical validation), for mining companies and government. Nevertheless, this study also recognizes the challenge of translating spatial evidence into policy, where despite quantitative analyses providing empirical evidence of injustices occurring and concentrated, they cannot capture the experiences of affected communities or what meaningful redress looks like in practice. Future research could, therefore, aim to use higher-resolution data enabling the detection of small-scale changes; include more time-reference points to show gradual transitions, consult more data sources such as subsurface data and socio-economic or household surveys; and entail comparative analysis across mining regions to strengthen findings and enable more comprehensive policy reform.

## Supplementary information

Supplementary information

## Data Availability

No restrictions apply to the availability of the land cover and administrative data. The original SANLC data presented in the study are openly available on the DFFE’s website at [http://egis.environment.gov.za/](https://egis.environment.gov.za). The administrative boundaries spatial data is available to the public from the Municipal Demarcation Board’s website at [https://spatialhub-mdb-sa.opendata.arcgis.com/](https://spatialhub-mdb-sa.opendata.arcgis.com). The pollution buffers data is available at [http://gisportal.gauteng.gov.za/portal/home/index.html](https://gisportal.gauteng.gov.za/portal/home/index.html). The mining lease areas data can be requested directly from the Department of Mineral and Petroleum Resources (formerly the Department of Mineral Resources). The corresponding author can be contacted to assist with the downloading of the data from online resources or facilitate access with the relevant departments if there are any challenges.
