# Supplementary material for: Mining Legacies and Environmental Injustice: Systematic Mining-Led LULC Change in the Witwatersrand Mining Belt, Gauteng, South Africa (1990–2020)
Source: Environ Manage. 2026 Oct 10;76(10):336. doi: 10.1007/s00267-026-02543-9 (PMC13653844; doi:10.1007/s00267-026-02543-9)
Supplement: Supplementary file 1 [file 267_2026_2543_MOESM1_ESM.docx]

**SUPPLEMENTARY INFORMATION:**

**Supplementary Table S1** LULC classes analyzed in this study

| **Collapsed LULC classes** | **Source LULC classes included** |
| --- | --- |
| Natural vegetation | Low forest and thicket, dense forest and woodland, wooded grassland, and natural grassland |
| Planted vegetation | Plantation forest, open and sparsely planted forest, temporary planted forest |
| Waterbodies and wetlands | Natural pans, artificial dams, herbaceous wetlands, and natural rivers |
| Barren land | Natural rock surfaces, dry pans, eroded lands, bare riverbed material, and other bare |
| Cultivated land | Cultivated commercial permanent orchards, commercial annual crops pivot irrigated, commercial annual crops non-pivot irrigated, commercial annual crops rain-fed/dryland, subsistence/small-scale annual crops, and fallow land, built-up formal (residential formal and roads and rail) |
| Formal built-up | Residential formal, residential formal (trees) |
| Informal built-up | Residential, informal residences and villages |
| Smallholdings | Smallholdings |
| Urban vegetation | Urban recreational fields |
| Commercial | Commercial |
| Industrial | Industrial |
| Mines and quarries, and waste | Mines, quarries and waste |

**Supplementary Table S2** LULC class persistence rates: Percentage of each LULC class remaining unchanged (1990-2020)

| **LULC class** | **Area of persisting pixels** | **Persistence rate (%)** | **Area changed pixels** | **Interpretation** |
| --- | --- | --- | --- | --- |
| Natural vegetation | 765.06 km² | 69.2% | 339.88 km² | Moderate stability, substantial loss |
| Cultivated land | 498.65 km² | 85.3% | 85.94 km² | High stability; agricultural resilience |
| Built-up formal | 268.77 km² | 74.7% | 91.25 km² | Moderate stability; expansion zones |
| Mines, quarries and waste | 81.82 km² | 56.7% | 62.42 km² | Low stability; active rehabilitation/expansion |
| Built-up informal | 2.30 km² | 45.4% | 2.76 km² | Highly dynamic; rapid expansion |
| Planted vegetation | 17.29 km² | 27.8% | 44.82 km² | Unstable; converted to other uses |

**Supplementary Table S3** Transition direction distribution: Over- and under-represented transitions

| **Transition type** | **Count** | **Percentage** | **Interpretation** |
| --- | --- | --- | --- |
| Over-represented (z-score > +2.0) | 29 | 20.6% | Class persistence dominates stability is more frequent than random, driven by systematic processes |
| Under-represented (z-score < -2.0) | 112 | 79.4% | Includes the major vegetation conversions, occurring less than random expectation |
| TOTAL SIGNIFICANT | 141 | 97.9% | High statistical power; observed patterns represent genuine landscape processes |

**Supplementary Table S4** Confidence intervals for key transitions (95% CI with binomial method)

| **Transition** | **Proportion (%)** | **95% ci lower** | **95% ci upper** | **Margin of error** |
| --- | --- | --- | --- | --- |
| Natural vegetation to Cultivated | 4.089% | 4.066% | 4.112% | ±0.023% |
| Natural vegetation to Built-up | 3.379% | 3.358% | 3.399% | ±0.021% |
| Mines to Natural vegetation | 2.088% | 2.072% | 2.105% | ±0.016% |
| Cultivated to Built-up formal | 1.431% | 1.417% | 1.444% | ±0.014% |
| Mining to Built-up areas | 0.031% | 0.029% | 0.033% | ±0.002% |
